# Interactions between *vitamin D receptor* (*VDR*) gene and *Interleukin-6* gene and environment factors on coronary heart disease risk in a Chinese Han population

**DOI:** 10.18632/oncotarget.19472

**Published:** 2017-07-22

**Authors:** Ma Jun, Guan Xue-Qiang, Li Jia, Xue Yang-Jing, Zheng Cheng, Jin Ge

**Affiliations:** ^1^ The Department of Cardiology, 2nd Affiliated Hospital of Wenzhou Medical University, Wenzhou 325000, China

**Keywords:** coronary heart disease, Interleukin-6, vitamin D receptor, single nucleotide polymorphisms, smoking

## Abstract

**Aims:**

To investigate the association of several single nucleotide polymorphisms (SNPs) within Interleukin-6 (*IL- 6*) and vitamin D receptor (*VDR*) gene, and additional gene- gene and gene- smoking interaction with coronary heart disease (CHD) risk in a Chinese population.

**Methods:**

Hardy-Weinberg equilibrium (HWE) examination was used by SNPstats (http://bioinfo.iconcologia.net/SNPstats). Generalized multifactor dimensionality reduction (GMDR) was used to screen the best interaction combination among SNPs and smoking. Stratified analysis for gene- smoking interaction was investigated by logistic regression.

**Results:**

CHD risk was significantly higher in carriers with the C allele of rs1800796 within *IL-6* gene than those with GG genotype (GC+ CC versus GG), adjusted OR (95%CI) =1.62 (1.19-2.23); CHD risk was also higher in carriers with the T allele of rs2228570 within *VDR* gene than those with CC genotype (CT+ TT versus CC), adjusted OR (95%CI) = 1.68 (1.26-2.17). However, we did not find any direct associations of the others SNPs in *IL- 6* and *VDR* gene with CHD risk. We also found a significant interaction between rs1800796 and smoking, the cross-validation consistency of this two- locus model was 10/ 10, and the testing accuracy was 60.11%. Current smokers with rs1800796- GC or CC genotype have the highest CHD risk, compared to never- smokers with rs1800796- GG genotype within *IL- 6* gene, OR (95%CI) = 2.57 (1.74 -3.46).

**Conclusions:**

We found that the C allele of rs1800796 within *IL-6* and T allele of rs2228570 within *VDR* gene, interaction between rs1800796 and smoking were all associated with increased CHD risk.

## INTRODUCTION

Coronary heart disease (CHD) is a major cause of death and disability worldwide [1]. In China, the CHD death rate increased from 141 per 10,000,000 person-years in 2003 to 229 per 10,000,000 person-years in 2010 [2, 3]. It has been well established that genetic and environmental factors, such as CHD family history, hypertension, diabetes mellitus, dyslipidemia, poor diet, advanced age and smoking habit, were associated with an increased risk of CHD [4].

CHD is associated with inflammation and considered as complex chronic diseases relating to multiple genes. And among these inflammatory cytokines, IL-6 is a circulating cytokine known as a regulation for inflammatory reaction, accelerates bone resorption [5] and plays a key role in atherogenesis and thrombosis [6, 7]. The *IL-6* gene is mapped to chromosome 7 at p21 [8]. Vitro or vivo studies have identified single nucleotide polymorphisms (SNPs) in the promoter regions of the IL-6 gene as principle mediators for the circulation of mRNA and *IL-6* levels and the status of inflammation [9, 10]. Several studies have reported the association between the *IL-6* gene SNPs and CHD susceptibility in different populations [11–13]. However, these studies could not conclude a certain and definitive conclusion. Several meta-analyses [14, 15] were also carried out attempting to compromise the existing inconsistencies with elusive results. The SNPs in vitamin D receptor (*VDR*) were found to be a potential risk factor of CAD, which might be associated with a low level of vitamin D in CAD patients, but the exact mechanism underlying the influence of *VDR* polymorphisms on the pathogenesis of CHD was still unknown [16–18]. CHD risk was influenced by not only genetic factors, but also some environment factors and gene- environment interactions, and in all modifiable risk factors, tobacco smoking was an important environmental risk factor for CHD. But to date, the relationship of *IL- 6* and *VDR* gene with CHD risk was not well known, and less study focused on the associations of gene- smoking interaction with CHD susceptibility. So the aim of this study was to investigate the impact of several SNPs within *IL-6* and *VDR* gene, and additional gene-smoking interaction on CHD risk, based on a Chinese population.

## RESULTS

A total of 1722 participants (880 males, 842 females) were selected, including 860 CHD patients and 862 control participants. The mean age of all participants was 63.8 ± 14.8 years. Table 1 shows the general characteristics in cases and controls. The mean of age and distributions of males and alcohol drinking were not significantly different between cases and controls. The means of TC, TG, LDL-C, BMI and WC, and the rate of smokers were higher in cases than controls. The mean of HDL-C was lower in cases than controls.

**Table 1:** General characteristics for all study participants in CHD cases and controls

Variables	CHD cases (N=860)	Controls (N=862)	*P-values*
Age (years)	63.5±13.6	64.2±14.1	0.295
Males N (%)	442 (51.4)	438 (50.8)	0.809
Drinking N (%)	221(25.7)	202(23.4)	0.275
Smoke N (%)	272 (31.6)	192 (22.3)	<0.001
WC (cm)	86.5±15.2	84.1±15.8	<0.001
BMI (kg/m^2^)	24.7±9.2	23.4±9.6	<0.001
TG (mmol/L)	1.5±0.7	1.3± 0.8	<0.001
TC (mmol/L)	4.8±1.2	4.2±1.3	<0.001
HDL-C (mmol/L)	1.16±0.4	1.31±0.5	<0.001
LDL-C (mmol/L)	2.51 ±0.72	2.71±0.76	<0.001
T2DM, N (%)	145 (16.9)	-	-
Hypertension, N (%)	338 (39.3)	-	-

WC, waist circumference; BMI, body mass index; N, number.

No significant difference in genotype frequencies from the Hardy–Weinberg equilibrium test was noted for any tested SNPs in the controls. The frequencies for the C allele of rs1800796 within *IL-6* and T allele of rs2228570 within *VDR* gene are significantly higher in CHD cases than controls (30.8% *vs*20.3%, 29.0% *vs*19.5%). Logistic regression analysis showed that CHD risk was significantly higher in carriers with the C allele of rs1800796 within *IL-6* gene than those with GG genotype (GC+ CC versus GG), adjusted OR (95%CI) =1.62 (1.19-2.23), and higher in carriers with T allele of rs2228570 within *VDR* gene than those with CC genotype (CT+ TT versus CC), adjusted OR (95%CI) = 1.68 (1.26-2.17). However, we did not find any direct association of the others SNPs in *IL- 6* and *VDR* gene with CHD risk after covariates adjustment. (Table 2).

**Table 2:** Genotype and allele frequencies of 6 SNPs between case and control group

SNP	Genotypes and alleles	Frequencies N (%)		OR (95%CI)*	*P*- values	*P-* values for HWE test in controls
		Control (n=862)	Case (n=860)			
***IL- 6* gene**						
rs1800795-174G/C						
	Co-dominant					
	GG	503 (58.4)	450 (52.3)	1.00 (ref)		0.994
	GC	311 (36.1)	338 (39.3)	1.26 (0.82-1.85)	0.287	
	CC	48 (5.6)	72 (8.4)	1.50 (0.77-2.31)	0.516	
	Dominant					
	GG	503 (58.4)	450 (52.3)	1.00 (ref)		
	GC+CC	359 (41.6)	410 (47.7)	1.34 (0.80-1.94)	0.489	
	Allele, C (%)	407 (23.6)	482 (28.0)			
**rs1800796-572G>C**						
	Co-dominant					
	GG	555 (64.4)	427 (49.6)	1.00 (ref)		0.118
	GC	264 (30.6)	337 (39.2)	1.38 (1.10-1.77)	0.0002	
	CC	43 (5.0)	96 (11.2)	2.18 (1.49-3.07)	<0.001	
	Dominant					
	GG	555 (64.4)	427 (49.6)	1.00 (ref)		
	GC+CC	307 (35.6)	433 (50.4)	1.62 (1.19-2.23)	<0.001	
	Allele, C (%)	350 (20.3)	529 (30.8)			
rs1800797-597G/A						
	Co-dominant					
	GG	494 (57.3)	441 (51.3)	1.00 (ref)		0.128
	GA	306 (35.5)	331 (38.5)	1.21 (0.80-1.78)	0.308	
	AA	62 (7.2)	88 (10.2)	1.45 (0.83-2.10)	0.672	
	Dominant					
	GG	494 (57.3)	441 (51.3)	1.00 (ref)		
	GA+AA	368 (42.7)	419 (48.7)	1.25 (0.80-1.87)	0.356	
	Allele, A (%)	430 (24.9)	507 (29.5)			
*VDR* gene						
*ApaI* (rs7975232)						
	Co-dominant					
	GG	499 (57.9)	445 (51.7)	1.00 (ref)		0.104
	GT	302 (35.0)	328 (38.1)	1.16 (0.78-1.63)	0.401	
	TT	61 (7.1)	87 (10.1)	1.56 (0.71-2.44)	0.532	
	Dominant					
	GG	499 (57.9)	445 (51.7)	1.00 (ref)		
	GT+TT	363 (42.1)	415 (48.3)	1.25 (0.76-1.89)	0.482	
	Allele, T (%)	424 (24.6)	502 (29.2)			
*FokI* (rs2228570)						
	Co-dominant					
	CC	564 (65.4)	442 (51.4)	1.00 (ref)		0.254
	CT	260 (30.2)	338 (39.3)	1.47 (1.22-1.85)	<0.001	
	TT	38 (4.4)	80 (9.3)	2.12 (1.43-2.88)	<0.001	
	Dominant					
	CC	564 (65.4)	442 (51.4)	1.00 (ref)		
	CT+TT	298 (34.6)	418 (48.6)	1.68 (1.26-2.17)	<0.001	
	Allele, T (%)	336 (19.5)	498 (29.0)			
*BsmI* (rs1544410)						
	Additive					0.115
	AA	548 (63.6)	484 (56.3)	1.00 (ref)		
	AG	269 (31.2)	301 (35.0)	1.30 (0.90-1.79)	0.213	
	GG	45 (5.2)	75 (8.7)	1.50 (0.81-2.31)	0.426	
	Dominant					
	AA	548 (63.6)	484 (56.3)	1.00 (ref)		
	AG+GG	314 (36.4)	376 (43.7)	1.36 (0.88-1.85)	0.384	
	Allele, G (%)	359 (20.8)	451 (26.2)			

*Adjusted for gender, age, smoking and alcohol status, BMI and WC.

We also investigate the impact of the interaction among 6 SNPs within *IL- 6* and *VDR* gene and gene- smoking interaction on CHD risk by using GMDR model. Table 3 summarized the results obtained from GMDR analysis. We found a significant two-locus model (p=0.0010) involving rs1800796 and smoking, indicating a potential interaction between rs1800796 and smoking on CHD risk. Overall, the cross-validation consistency of this two- locus model was 10/ 10, and the testing accuracy was 60.11%. We also conducted stratified analysis for the significant models in the GMDR analysis by using logistic regression. We found that current smokers with rs1800796- GC+CC genotype have the highest CHD risk, compared to never- smokers with rs1800796- GG within *IL- 6* genotype, OR (95%CI) = 2.57 (1.74 -3.46), after covariates adjustment (Table 4).

**Table 3:** GMDR analysis on the best gene–gene and gene- smoking interaction models

Locus no.	Best combination	Cross-validation consistency	Testing accuracy	*P-values*
Gene-gene interactions^*^				
2	rs1800796 rs2228570	8/10	0.5399	0.0547
3	rs1800796 rs2228570 rs1800797	7/10	0.5399	0.1719
4	rs1800796 rs2228570 rs1800797 rs1544410	6/10	0.5399	0.3770
5	rs1800796 rs2228570 rs1800797 rs1544410 rs7975232	6/10	0.4958	0.4258
6	rs1800796 rs2228570 rs1800797 rs1544410 rs7975232 rs1800795	5/10	0.4958	0.6230
Gene- smoking interactions ^**^				
2	rs1800796 Smoking	10/10	0.6011	0.0010
3	rs1800796 rs2228570 Smoking	8/10	0.5399	0.0547
4	rs1800796 rs2228570 rs1800797 Smoking	7/10	0.5399	0.1719
5	rs1800796 rs2228570 rs1800797 rs1544410 Smoking	7/10	0.4958	0.3770
6	rs1800796 rs2228570 rs1800797 rs1544410 rs7975232 Smoking	6/10	0.4958	0.4258
7	rs1800796 rs2228570 rs1800797 rs1544410 rs7975232 rs1800795 Smoking	4/10	0.4958	0.6230

*Adjusted for gender, age, smoking, drinking and BMI for gene- gene interaction analysis.

**Adjusted for gender, age, drinking and BMI for gene- smoking interaction analysis.

**Table 4:** Stratified analysis for gene- smoking interaction by using logistic regression

rs1800796	Smoking	OR (95% CI)^*^	*P-values*
GG	Never	1.00	-
GC+CC	Never	1.33(1.02 -1.76)	0.032
GG	Current	1.46 (1.15-1.85)	0.001
GC+CC	Current	2.57 (1.74 -3.46)	<0.001

*Adjusted for gender, age, drinking and BMI.

## DISCUSSION

In this study, we found that both the C allele of rs1800796 within *IL-6* and T allele of rs2228570 within *VDR* gene were significantly associated with increased CHD risk. Several similar studies have been performed on association between *IL-6* SNP and CHD risk; however these studies concluded inconsistent results. Gigante [19] indicate that IL6R haplotypes regulate the circulating levels of inflammatory biomarkers. But lack of association with the risk of CHD may be explained by the combined effect of SNPs with opposite effect on the CHD risk. A meta- analysis conducted by Song et al [20] suggested that the *IL-6* -572G>C polymorphism may be linked with risk of CHD in a protective model. Another Chinese study [21] indicated that the -572G/C polymorphism in the *IL-6* gene promoter region was involved in the pathogenesis and progression of CHD in the Han Chinese. But some other studies concluded different results with these studies. Tong et al [22] indicated that *IL-6* SNPs were associated with increased CAD risk in the Chinese population, and may be useful predictive markers for CAD susceptibility. A meta- analysis [23] indicated that *IL-6* gene −174 G/C polymorphism is associated with increased CHD risk among Asians. Another Chinese case- control study [24] also suggested that *IL-6*-572C/G polymorphism was associated with susceptibility to CHD after adjustment for other risk factors such as smoking, hypertension, hyperlipidema, obesity and alcohol drinking, they concluded that *IL-6*-572C/G polymorphism might be a potential risk factor for CHD in Chinese population. The potential mechanism for the association between *IL-6*-572C/G and CHD was not well known, but studies have indicated that inflammation plays an important role in the progression of atherosclerosis [25]. IL-6 functions as a multifunctional cytokine, playing a critical role in the inflammatory response in relation to the etiology of CHD [26].

The gene encoding the *VDR* is located at chromosome 12q and has 4 common polymorphisms. *BsmI* (rs1544410), *ApaI* (rs7975232) and *FokI* (rs2228570) were three common SNPs in *VDR* gene polymorphism. More and more recent attention has been focused on the possible role of *VDR* gene polymorphisms in the development of a range of diseases, including diabetes [27], as well as CAD [28–30]. Some previous studies also concluded inconsistent results on association between *VDR* gene and CHD risk. Pan et al [18] indicated that there was no significant difference in the genotype distribution or the allele frequencies of *VDR FokI* and *BsmI* between case and control groups in a Chinese population. Another study [31] also indicated that low vitamin D level was associated with an enhanced risk for incident CAD, but *VDR* genotypes did not show any association with either vitamin D levels or CAD. However the opposed results indicated that the haplotype comprising the minor allele of *BsmI*, major allele of *ApaI* and minor allele of *TaqI* of *VDR* (*AAC*) was associated with an increased risk of CAD in type 2 diabetes patients [30]. A meta- analysis [29] suggested that the *Fok1* polymorphism may play a protective role in CAD, and the possible protective role in *Apa1* CA genotype in CAD patients with T2DM still needed more studies to support. The *Taq1* polymorphism is found to be associated with a significant increase in CAD risk based on our analysis. Moreover, increased risk in *Apa1* variation in CAD patients without T2DM and *Bsm1* variation in Caucasian group is also detected. But another meta- study [16] concluded that *VDR FokI* polymorphisms appear to be associated with CHD, but *Apa1* and *Taq1* was not associated with CHD risk. The SNPs in VDR were found to be a potential risk factor of CAD, which might be associated with a low level of vitamin D in CAD patients, but the exact mechanism underlying the influence of VDR polymorphisms on the pathogenesis of CAD is still unknown. Several frequent polymorphisms in the VDR gene have been reported to be associated with a variety of physiological and pathological phenotypes, including intrauterine and early postnatal growth, body weight and body height, as well as insulin secretion, insulin sensitivity, glucose tolerance and susceptibility to both type 1 and type 2 diabetes [32–34].

CHD susceptibility was influenced by both genetic and environmental factors, and previously several environmental factors associated with CHD have been reported, and in these risk factors, cigarette smoking has been suggested to play a crucial role in increasing the risk of CHD risk. In current study, the rate of smoking was higher in CHD cases than controls, so in this study we not only investigated gene- gene interaction on CHD, but also gene- environment interaction between SNPs and smoking. We found a significant interaction between rs1800796 and smoking on CHD risk, current smokers with rs1800796- GC or CC genotype have the highest CHD risk, compared to never- smokers with rs1800796- GG within *IL-6* genotype.

There several limitations in our study. Firstly, more SNPs within *IL-6* and *VDR* gene should been included in the analysis, not only for just 6 SNPs. Secondly, some others environmental risk factors should be investigated in the gene- environment interaction analysis. Thirdly, haplotype analysis should be performed in the future studies. Lastly, both the CHD and control groups were recruited from one hospital, and the controls were selected using convenient sampling. These selected controls could not represent the general population, because these people received physical examination routinely pay more attention to their health.

In conclusion, we found that the C allele of rs1800796 within *IL-6* and T allele of rs2228570 within *VDR* gene, interaction between rs1800796 and smoking were all associated with increased CHD risk.

## MATERIALS AND METHODS

### Subjects

**Figure 1:**
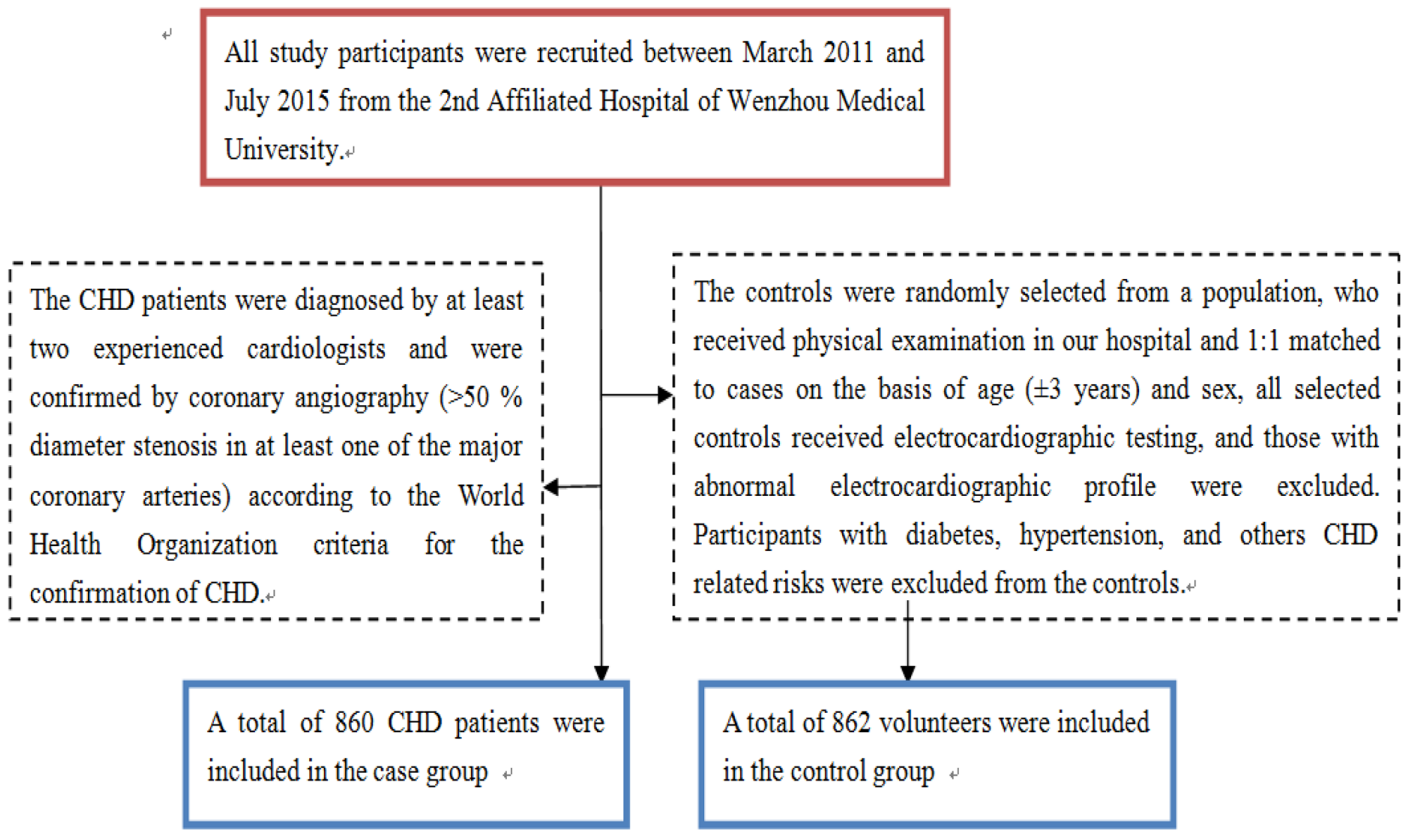
A flowchart on study population selection and exclusion

All study participants were recruited between March 2011 and July 2015 from the 2nd Affiliated Hospital of Wenzhou Medical University. The CHD patients were diagnosed by at least two experienced cardiologists and were confirmed by coronary angiography (>50 % diameter stenosis in at least one of the major coronary arteries) according to the World Health Organization criteria for the confirmation of CHD. The controls were randomly selected from those, who received physical examination in our hospital and 1:1 matched to cases on the basis of age (±3 years) and sex, all selected controls received electrocardiographic testing and coronary angiography, and those with abnormal electrocardiographic and coronary angiography profile were excluded. Participants with diabetes, hypertension, and others CHD related risks were excluded from the controls (Figure 1). Both the CHD cases and controls were unrelated Han Chinese population. Questionnaire investigation was conducted for all participants, and data on demographic information, clinical and biochemical index for all participants were obtained. Body weight, height and waist circumference (WC) were measured. Current cigarette smokers were those who self-reported smoking cigarettes at least once a day for 1 year or more, and still smoked at the time of the interview, and individuals with no history of cigarette smoking were considered as never smokers. Alcohol consumption was expressed as the sum of milliliters of alcohol per week from wine, beer, and spirits. Blood samples were collected from each participant in the morning after at least 8 hours of fasting. Informed consents were obtained from all participants.

### Genomic DNA extraction and genotyping

The SNPs were selected based on the NCBI database (http://www.ncbi.nlm.nih.gov/projects/SNP), those SNPs with a minor allele frequency (MAF) of at least 5%, which have been reported in previous studies, but were not well studied, were selected for genotyping, including: rs1800796, rs1800797 and rs1800795 within *IL-6* gene; rs2228570, rs1544410 and rs7975232 within *VDR* gene. Genomic DNA from participants was extracted from EDTA-treated whole blood, using the DNA Blood Mini Kit (Qiagen, Hilden, Germany) according to the manufacturer’s instructions. Genotyping for all 6 SNPs was performed by polymerase chain reaction (PCR) restriction fragment length polymorphism (PCR-RELP) method. The specific primers for the 6 SNPs listed in Table 5.

**Table 5:** Description and probe sequence for 6 SNPs used for PCR analysis

SNP ID	Chromosome	Functional consequence	Major/ minor alleles	Restriction enzyme	Primer sequences
***IL- 6* gene**					
rs1800795-174G /C	7:22727026	Intron variant, upstream variant 2KB	G/ C	*NlaIII*	F: 5’- tgacttcagctttactctttgt-3’ R: 5’- ctgattggaaaccttattaag-3’
rs1800796-572G>C	7:22726627	Intron variant, nc transcript variant, upstream variant 2KB	G / C	*BsrBI*	F: 5’- gagacgccttgaagtaactg-3’ R: 5’- aaccaaagatgttctgaactga-3’
rs1800797-597G/A	7:22726602	Intron variant, nc transcript variant, upstream variant 2KB	G/ A	*FokI*	F: 5’- ctcctctaagtgggctgaag-3’ R: 5’- caagcctgggattatgaaga-3’
***VDR* gene**					
FokI (rs2228570)	12:47879112	Missense	C/ T	*FokI*	F: GCACTGACTCTGGCTCTGAC R: ACCCTCCTGCTCCTGTGGCT
BsmI (rs1544410)	12:47846052	Intron variant, upstream variant 2KB	A/ G	*FspI*	F: GGAGACACAGATAAGGAAATAC R: CCGCAAGAAACCTCAAATAACA
ApaI (rs7975232)	12:47845054	Intron variant, upstream variant 2KB	G/ T	*ApaI*	F: TGGGCACGGGGATAGAGAAG R: ACGGAGAAGTCACTGGAGGG

Genotyping for rs1800796, rs1800797 and rs1800795 within *IL-6* gene: a 25 μl reaction mixture was amplified by PCR, containing 100 ng genomic DNA, 1.5 mM MgCl_2_, 0.2 mM dNTPs, 1 mM each primer, reaction buffer, and 0.625 U Taq polymerase. PCR conditions were as follows: initial denaturation at 94^°^ C for 3 min, followed by 35 cycles of 94°C for 30 s, 56° C for 30 s (52° C for 30 s for rs1800795), 72° C for 20 s, and a final extension of 72° C for 5 min. Genotyping for rs2228570, rs1544410 and rs7975232 within *VDR* gene: The reaction volume was 25μl, containing Taq DNA polymerase, dNTPs mix and reaction buffer (Shanghai, China). A total of 0.5 μM of each primer, 2μl of template cDNA, 9μl 2.5* Mastermix, and 8.5 μl distilled water, (ddH_2_O) were used in all reactions (in triplicate). PCR conditions were as follows: denaturation 95 ° C, 15 s; annealing 60° C, 60 s; elongation 72° C, 45 s) consisted of 40 cycles to ensure a linear range of the PCR reaction.

### Statistical analysis

All statistical analyses were performed using the SPSS 22.0 software package (SPSS Inc, Chicago) for Windows 7. Categorical variables were presented as absolute values and percentages, and continuous variables were expressed as mean ± standard deviation (SD). Student’s t test was used to compare continuous variables, while Chi-square test was used to compare categorical variables between cases and controls. Hardy-Weinberg equilibrium (HWE) examination was used by SNPstats (http://bioinfo.iconcologia.net/SNPstats). Generalized multifactor dimensionality reduction (GMDR) model was used to analyze the gene- gene interaction, some parameters including cross-validation consistency, the testing balanced accuracy and the sign test were calculated, a sign test or a permutation test (providing empirical p-values) for prediction accuracy can be used to measure the significance of an identified model. Logistic regression was performed to investigate association between 6 SNPs within *IL-6* and *VDR* gene and CHD, and additional stratified analysis for gene- smoking interaction on CHD risk. Two sided test with *P* < 0.05 was considered statistically significant.
